# Animal welfare chauvinism in Brexit Britain: a genealogy of care and control

**DOI:** 10.1057/s41292-022-00282-8

**Published:** 2022-09-10

**Authors:** Reuben Message

**Affiliations:** School of Geography and the Environment, University of Oxford, Oxford, United Kingdom

**Keywords:** Brexit, Britain, Nationalism, National cultures of care, Animal welfare, Genealogy

## Abstract

This paper uses the deployment of animal welfare as an issue during the ‘Brexit’ referendum as a lens through which to explore the mutual shaping of discourses about care for animals in Britain and the British nation, or the nationalism of animal welfare. Adopting a genealogical outlook, it uses one political advertisement in particular—paid for by the official Vote Leave campaign—as a focalising image and means of opening up the issues, leading to an empirical emphasis on the issue of live animal export as it has mediated ideas about Europe and British identity. Introducing the idea of ‘animal welfare chauvinism’, the paper suggests that animal welfare messages in the context of this constitutional debate were products of chauvinistic and caring impulses which are mutually constitutive and crystallised through discourses formed in relation to contingent historical struggles. Analytically, stress is placed on the constructive role of situated and repeated discursive exchanges, occurring between animal advocates and other national political elites, within which ‘care for animals’ as a national ideal is forged. In this light, the article concludes with reflections on the stakes of entering into an already existing conversation on the ‘national culture of care’ for animals in Britain.

## Introduction

**Fig. 1:**
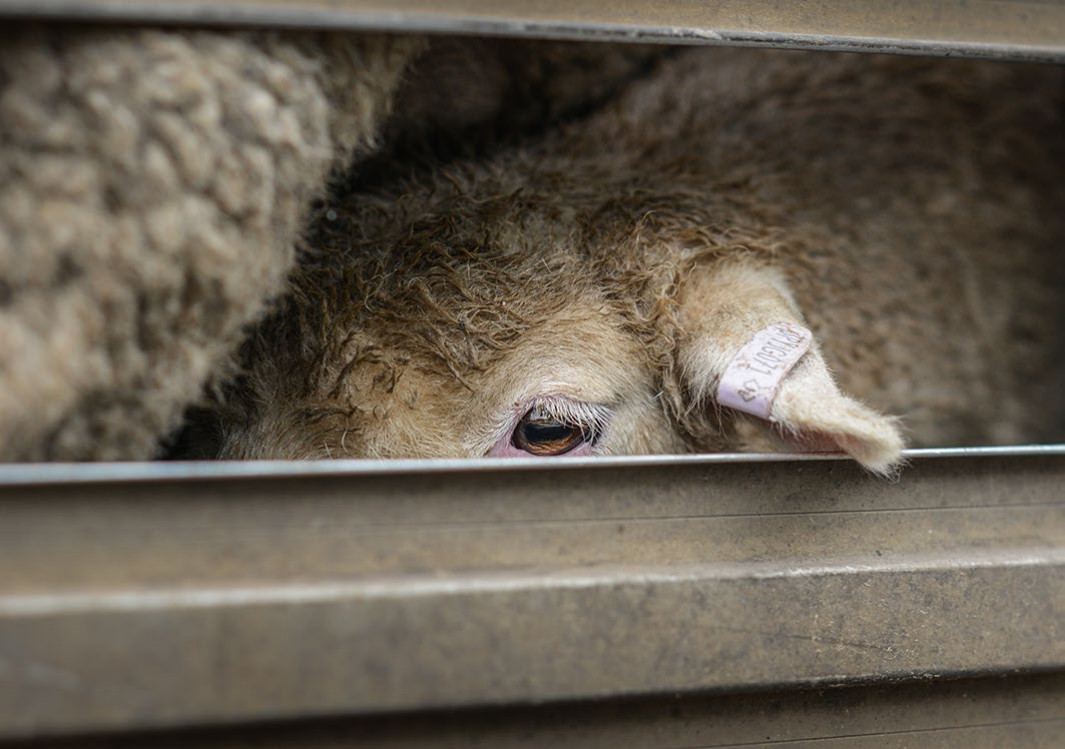
This image was appropriated and used as a part of Vote Leave’s Brexit referendum campaign following earlier use online by animal welfare groups. Reproduced with permission of Jo-Anne McArthur/We Animals Media (https://weanimalsmedia.org/)

In May 2016, an advertisement appeared on the newsfeeds of millions of British Facebook users. It featured the resigned face of a sheep in transit. A powerful rendition of a familiar trope, anyone who saw the image knew: this was a sheep bound for slaughter. The photograph used in this advertisement was originally taken by a photojournalist and animal advocate in 2013 at the sale yards in Ballarat, Australia (Fig. 1).^1^

In this case, however, the image was being used a part of Vote Leave’s online micro-targeting campaign during the Brexit referendum. Echoing their famous slogan “Take Back Control”, words emblazoned across the photograph at the time of its publication on Facebook asked the rhetorical question: “Shouldn’t we be in control of our animals and their welfare?”. Accompanying text claimed that the EU does not believe that transporting livestock for slaughter is inhumane, and that banning the practice is illegal under European law. By thus identifying both the subservience of British law to European law, and ‘Europe’ with animal cruelty, the advertisement concisely recapitulated a central Eurosceptic argument that reclaiming legal sovereignty from Europe would result in greater substantive sovereignty for Britain, and thus Britain’s ability to act upon its own values and interests.

Appropriated and recontextualised in this way, the image embodied a distinct history and set of meanings. As an advertisement, moreover, it was only one of a family of animal welfare-themed advertisements placed by the digital strategy consultancy AggregateIQ on behalf of Vote Leave around this time.^2^ Others featured polar bears on melting ice floes, whales being butchered, and a bloody bullfighting scene. Collectively, by means of contrast to Europe, they presented Britain as nation that cares for animals, and in so doing participated in a long-established genre of representations in which abhorrence of the treatment of animals by foreigners acts as an “index of Britishness” (Baker 1993, p. 63). Commenting on them in a radio interview a while later, Thomas Borwick, Chief Technology Officer of the official ‘Leave’ campaign, claimed that these ads actually outperformed bona fide ‘big issues’ like immigration and the National Health Service as online electioneering events. Helped by its clear identification of an enemy, he said, an image of “a sheep being taken to the slaughter, because of the rules and regulations that we currently have in place, is a very, very powerful message” (Analysis 2018). There should be little wonder, then, that during his first address to the nation as Prime Minister, Boris Johnson declared it a priority of his government to “promote the welfare of animals that has always meant so much to the British people” (Johnson 2019). Thus, while animal welfare only ever constituted a tiny portion of the issues debated during the campaigns associated with Brexit, it was nevertheless a powerful one.

Vote Leave’s ad was, like all political advertising, a manipulative device, and the technological segregation of society into micro-constituencies it’s deployment embodied is, arguably, a tactic fit only for a divided, post-ideological society. But in the context of a national constitutional revolution, it can also be thought of as a claim about Britain’s ‘national culture of care’ for animals—and, consequently, a way of laying claim to it. In this light, when the very question of British identity appeared to be at stake, the ad felt emblematic of a form of narrative in which patriotism and national belonging are expressed in terms of care for animals and associated values. The ad, indeed, was so potent because it mixed powerful national myths. On the one hand, it implied that the British embody a distinctive moral sensitivity towards animal suffering. On the other, less obviously perhaps, it was steeped in a longstanding conviction, central to the idea of the British state, that the British are also a uniquely free people—“‘Britons never will be slaves’” as the patriotic anthem goes—and that their freedom is a sign of their virtuousness and a condition of their humanity (c.f. Colley 1992). Without sovereignty, Vote Leave therefore suggested, Britain is party to inhumanity. Relaying the relevance of this historically old compound of sovereignty and humanity into contemporary rhetoric, Boris Johnson (then leading campaigner for Vote Leave) argued in his ‘victory speech’ the day after the referendum that “[a]bove all we can [now] find our voice in the world again. A voice that is commensurate with the fifth biggest economy on earth—powerful, liberal, humane—an extraordinary force for good in the world” (quoted in Hope 2016). Yet it should also be obvious that these ideas contain inherent contradictions: humanitarian outcomes often cannot be achieved without constraints on the exercise of individual liberty.^3^

Regularly described in terms of national ‘rejuvenation’, ‘crisis’ or ‘decline’, Brexit no doubt surfaced many questions about British identity and history, the country’s putative exceptionalism and its constitutional settlement, all of which felt unusual in their urgency and salience (e.g. Abulafia 2015; Nedergaard and Henriksen 2018; Various Authors 2015). Thus, I think, Brexit also represents an opportune moment at which to reconsider the mutual shaping of discourses about care for animals and the British nation. To this end, this paper offers an (inevitably) incomplete and partial genealogy of Vote Leave’s ‘sheep-to-the-slaughter’ advertisement. Through this, it gives an account of the nationalism of animal welfare in Britain, or what I will call 'animal welfare chauvinism'. In doing so, it presents an occasion to re-evaluate some of the constitutive social values that shape how care for animals is understood, spoken about and instrumentalised today.

## Historiography to genealogy

The circumstances shaping British attitudes to animals have received much attention from historians (classics include Harwood 2002; Thomas 1984; Turner 1980, 1992). In this context, scholars have proposed various connections between ideas of ‘the nation’ and animal welfare. It is for example noted that Evangelicals viewed animal protection as a part of their wider campaign to change “the British national image” by ridding God’s nation of sin and vice (Harrison 1973, p. 802). For Tague (2015, p. 172), care for animals became a symbol of “a broader humanitarianism” that was brought increasing into the “centre of Britain’s national identity” during the late eighteenth century. Drawing on Colley’s (1992) account of the religious and cultural glue behind the unified British state, Ferguson (1998) emphasised how this new humanitarianism developed in the context of national humiliation following the loss of the American war and then decades of strife with Revolutionary and Napoleonic France. While the British ruling classes remodelled their image in terms of conspicuous public service and humanitarian action, Ferguson emphasised how some socially elite women in particular found that care for animals could be a way of both expressing patriotism and entering public life that was appropriate to their circumscribed social station and consonant with the national mood. Moreover, it is widely noted in this literature that British caring has always been underscored by contrasting it with the supposed cruelty of her geopolitical rivals, and mixed in with criticism of their modes of government and religious beliefs: indeed, xenophobic and especially anti-Catholic sentiment was a regular feature of early animal protection discourse (see especially Recarte 2014). Indeed, animal protectionism has even been viewed by some as synonymous with ‘bourgeois morality’ during the Victorian era, and consequently a part of the apparatus by which the bourgeoisie shaped the nation in its image (also Boddice 2005; Tester 1991, p. 118).

In this paper, I both build on and depart from these outlooks. I agree with Ritvo that the connection between national character and kindness to animals was initially forged principally as a “rhetorical strategy” promoted by animal advocates (Ritvo 1989, p. 129). But I think that greater attention needs to be paid to the discursive strategies of other parties too, notably those associated with national politics. It is of course well known that British politicians have a special penchant for being photographed with animals: it is said to help ‘soften’ their image before the public (Barkham 2015). In doing so though, they vie to embody a cherished idea of the nation, and consequently help perpetuate it. As such, a comprehensive account of how Britain is continuously reproduced as an ‘animal loving nation’ requires explicit focus on the discursive exchanges and subtle quid pro quos that occur between animal advocates and other national elites, and the role of these elites *as* animal advocates. To begin doing this, however, this paper departs from the historiographical orientations established by scholarship on the eighteenth and nineteenth centuries, and adopts a genealogical outlook inspired by Foucault’s approach to history. I take this, for all its lack of methodological systematicity, to centrally involve the “uncovering of hidden conflicts and contexts as a means of re-valuing the value of contemporary phenomena” (Garland 2014, p. 365), especially where these are normally taken for granted: that is to say, examining through discourse the specific power struggles and instrumentalisations that help to create our collective moral common sense.

Genealogy by nature does not lend itself to tightly drawn sampling frames and, correspondingly, the materials used in this analysis are heterogenous and distributed in time. They include articles from periodicals, news sites and established national newspapers, official reports and policy statements from government departments and charities, as well as parliamentary proceedings. Indeed, a part of the challenge of investigating ‘national’—or, at least, ubiquitous—discourses in the present or very recent past is the sheer quantity of sources. To help ensure breadth, however, I used research on media bias during the referendum campaign to guide my initial sampling. Conducted by the Reuters Institute for the Study of Journalism at Oxford (Levy et al. 2016), this research enabled me to rank national newspapers in terms of bias with respect to reporting on the 2016 referendum, and then use this ranking as a way ‘checking’ that I was reading across a wide range of views.^4^ Additionally, I paid extra attention to reporting in leading periodicals, especially *The Spectator* and *New Statesman,* two titles traditionally considered to represent the centre right and centre left of national political discourse respectively. From this spine, I used conventional methods of desktop and historical research to branch out, follow leads and research specific themes and stories that I judged would best illustrate the lines of descent and transformation of interest. For the more historical sections of the paper—namely those on live animal export in the context of European integration—I relied mainly on the online archives of two leading nationals from differing parts of the political spectrum, namely the *Guardian* and *Times.* Overall, and while the easiest exemplars of the discourses in question may often be found at the extremes, I suggest that this approach helps maintain a focus on uses of language that are widely shared, and, in this sense, broadly ‘national’ in character.

## Animal welfare chauvinism and the Brexit campaigns

Animal welfare issues not only animated ‘secretive’ online campaigning during the Brexit referendum but also formed a strong current of general media debate about what leaving the EU would mean, with both sides predicting dire consequences for animals should the other side win. Some of this was measured debate about an important series of policy trade-offs (McCulloch 2018, 2019). As the RSPCA estimated, because so much activity involving animals impinges on the functioning of the European Single Market, approximately 80% of UK animal welfare regulations were indeed based on EU laws at this time (RSPCA 2016). Consequently, however, any intervention into this debate was inevitably deeply political. Generally, ‘Remainers’ highlighted the risk of falling standards upon leaving the EU; ‘Leavers’ pointed out problems with the status quo; and formally neutral animal advocacy groups tended to do both. All, however, were indebted to an identifiable, widespread linguistic construct of some lineage. I call this ‘animal welfare chauvinism’. In this section, therefore, I briefly situate Vote Leave’s ‘sheep-to-the-slaughter’ advertisement in its immediate rhetorical context.

Animal welfare chauvinism simply holds that the British are peculiarly compassionate towards animals—a thoroughly banal form of nationalism indeed (see Billig 1995; Swart 2018). Its most quotidian—and seemingly ahistorical—embodiment is surely that ubiquitous expression of national togetherness: the idea that Britain is a ‘nation of animal lovers’. Animal welfare chauvinism further implies that British animal welfare standards are both the result of and corroborating evidence for this distinctive national characteristic. It thus lends itself naturally to claims about international leadership in the field of animal humanitarianism, from whence also follows the claim that adherence to European welfare standards would be morally dangerous. Highlighting differences between national communities in the way animals are treated is fundamental to its effective functioning and, as such, its identification of ‘constitutive others’ also contributes to the continuous construction of Britain as an “imagined political community” (Anderson 2006), whatever the specific intentions of those engaging in these rhetorical performances may be. I interpret animal welfare chauvinism therefore as a shared resource, a flexible construct that is co-created and reproduced in exchanges by different agencies as it is mobilised towards different ends by different groups. In the situated interplay of their interests, non-partisan animal advocates and other national political elites mutually sustain and co-constitute animal welfare chauvinism and the linguistic devices, tropes, images and topics that characterise it. Importantly, there is thus no reason to commit the historiographical error of reducing the activity of animal advocates who deploy the rhetoric of animal welfare chauvinism to underlying or veiled socio-political (e.g. nationalistic) motives in making this case: we can see them as creative opportunists, strategic agents deploying language selectively to their own ends within the constraints of context (see discussions in Donald 2020, pp. 3–4; Li 2019). Thus, I assume that Brexit activated animal welfare chauvinism, but certainly never invented it.^5^

In its construction, the term ‘animal welfare chauvinism’ resembles Andersen and Bjørklund’s (1990) well-known idea of “welfare chauvinism”, a phrase used to describe the tendency of European right wing parties’ desire to restrict the benefits of the welfare state to select ‘deserving’ groups. As discussed below, animal welfare chauvinism was sometimes deployed during the Brexit campaigns towards similar or compatible political ends. But this resemblance should not con us into believing in any deeper conceptual coherence between the terms. Indeed, animal welfare chauvinism in Britain, as we will see, does not hold that welfare should only be extended to a certain category of ‘deserving’ (i.e. British) animals: to the contrary, it may be found in the perennial popularity of initiatives to extend British care to suffering animals in foreign climes.

An article entitled “Voting in? You have the blood of Spanish bulls on your hands” in the Brexit-supporting periodical the *Spectator* represents a convenient exemplar. Published in June 2016, the month of the referendum, it’s author argued that the Britons “have always had a strange relationship with animals”, and claimed that this explains “why we have some of the highest animal welfare standards” today (Swift 2016). Indeed, it declared, “we care more about animal welfare” than people in the EU do. Referring to foie gras production, for example, it noted that the French “prize gastronomy far above husbandry”. The EU, it argued, does nothing to protect animals. Thus, leaving the EU would “let us make the most of our reputation as animal lovers”. Instead of “compromising our standards we could brand ourselves as a beacon of higher-welfare farming—and set an example not just to Europe but to the world” (Swift 2016).

There is nothing unusual about these assumptions or phraseology—indeed, much of it is entirely cliché in British public discourse. Clearly it is the stuff of that most British of institutions, the tabloids (c.f. Baker 1993). But it is also found in the lobbying activities of animal advocates. After the referendum, an opportunistic advertisement placed in the *Spectator* was bound to attract attention. On the 16th of December 2017, just three days after the government lost a crucial vote on the EU Withdrawal Bill, a small charity that specialises in directing funds to causes in Europe highlighted the plight of stray dogs in Romania under the headline “Roll on Brexit. These animals are dying to get out of Europe”.^6^ Drawing attention also to the perennial scandals of foie gras and bullfighting, it explained that “Brexit means that the UK can set an example of animal welfare that is long overdue in Europe”. (Notably, the claim that Britain indirectly subsidised bullfighting in Iberia via the Common Agricultural Policy was also well excised during the Brexit referendum campaign, see e.g. *BBC *News 2016).

As a charity, Burnie’s Foundation is unusual for its explicit endorsement of a partisan cause—but the animal welfare chauvinism “Roll on Brexit” it exemplified is not. The director of PETA in Britain, for example, recently claimed that Brexit represents an “opportunity” that must be finally seized, and foie gras, a most “un-British product of torture”, made to disappear from these shores (Allen 2019). As she pointed out, the moral inconsistency whereby foie gras production is banned in the UK while its sale or importation from the Continent is allowed is largely an artefact of EU Single Market policies. Animal welfare chauvinism is even reflected in the soberer language of other policy influencers. An agenda setting paper by a coalition of well-established animal advocacy groups claimed that with Brexit “[w]e have a once in a lifetime opportunity to either define or undermine our country’s identity and reputation as a global leader in animal welfare science and standards” (*Brexit: Getting the best deal for animals*, 2018, p. 5). It is similarly discernible in the language of those promoting the adoption of UK animal welfare standards and technologies globally (see Davies 2019).

A strategic affinity between ‘Leavers’ and the rhetoric of animal welfare chauvinism may be clear. But it’s important to emphasise that it framed the language available to ‘Remainers’ too. They also mostly assumed British superiority in this sphere, arguing consequently that staying part of Europe would allow Britain to continue to influence the standard of EU welfare provisions. During a controversial public debate on ‘animal sentience’ in November 2017—a debate concerning whether or not to transpose into UK law the Treaty of Lisbon’s explicit recognition that animals are sentient—the leading role that British organisations and officials played in developing the relevant protocol in the 1990s was cited as a source of pride by both sides of the debate and as evidence of international moral leadership (Vasudhevan 2019). Earlier, in April 2016, a letter to the *Guardian* signed by a cross-party group of Remain-supporting MPs urged “everyone who cares about animals to vote Remain”, arguing:

Animal advocates know that EU rules on animal protection don’t go nearly far enough, but to improve the standards, we need to remain part of the EU and strive to make them stronger. On some critical animal protection issues, […] the UK has actually shown leadership in the EU; we have played a full part in shaping EU-wide standards, and should continue to do so (Letters 2016).

The assumption—that Britain enlightens morally benighted Europeans—is familiar and shared. This said, animal welfare chauvinism was usually a more unreliable ally for ‘Remainers’ than ‘Leavers’. An article in the *Guardian* a week earlier noted that, despite Britain having “a reputation as a nation of animal lovers”, it currently needed “continued European Union input”, as it had come to rely on EU law to “[protect] British animals from cruel farming practices” (Barker 2016). This is effectively to suggest that Britain, despite its undoubted compassion for animals, needed help from Europe to protect its animals from itself. This strikes at the core of animal welfare chauvinism—indeed, in the parlance of the era, was tantamount to ‘talking the country down’. Where animal welfare chauvinism began to benefit ‘Remainers’ was really only after the referendum, when attention turned towards arguing about the trade-offs of future trade deals. In this context, US-produced ‘chlorine washed chicken’, for example, became an overarching symbol of the anxieties triggered by the prospect of leaving the EU’s regulatory sphere, as well as a stick to beat Brexiteers with (see e.g. Bush 2017; Elgot 2017). Would British producers need to lower their welfare standards in order to compete? Identifying a new external threat or ‘other’—a trade regime dominated by the USA—animal welfare chauvinism would chime better with a pro-European agenda and, in its own turn, bury the contradiction between animal humanitarianism and the market liberalism that being party to the EU’s major economic institutions entails.

## Live animal export and the question of Europe

During the ‘extended’ campaign that characterised the Brexit debate (that is, both the official campaigning period and the period of political turmoil that followed the referendum result) Brexit was regularly presented as an ‘opportunity’ for improving the lot of many kinds of creatures, including ducks, whales, puppies, bulls used in bullfighting, and experimental animals (see e.g. Ferguson 2017; *The *Telegraph 2018; Winter 2016, 2017). But ‘Exhibit A’ is undoubtedly farm animal welfare and live animal export in particular. This is also the key reference point for understanding Vote Leave’s sheep-to-the-slaughter advertisement.

A fixture in animal advocacy, the issue of live exports tends to breach into the public consciousness with vehemence in relation to specific events. Very often these are associated with political divisions vis-à-vis European supranational economic and agricultural institutions. This was the case on September 6th 1990, when, in the French market town of Bellac, what *The Times* described as an “angry mob” of 200 farmers “ambushed” a truck filled with 386 sheep of British origin using a barricade of burning tyres. Later, they marched the animals to the town’s abattoir for slaughter—although other reports claimed that the lambs, between five and six months old and sent to France for fattening, were really burnt alive. In another incident, a lorry was “hijacked” in Lyon, with 200 animals being released onto the streets, later to be killed, some of them by burning (Hornsby 1990b). Reports of these incidents and others, known as the ‘lamb wars’, circulated widely in the UK and were often accompanied by harrowing images of distressed sheep and thuggish French farmers.

These events were widely understood as fallout from the failure of European Community (EC) agriculture reforms in the 1980s to limit a collapse in the price of sheep meat, a problem caused largely by rapid industry over-expansion funded by EC subsidies. French sheep farmers suffered particularly from this and they aired their grievances by attacking consignments from their more efficient and better supported British counterparts (about 75% of whose sheep meat exports went to France at this time) (Hornsby, 1990c, d). British farmers, in turn, used the crisis to draw attention to the EC subsidies received annually by French farmers on a per ewe basis, though their real concern was probably the impending threat of losing a price support mechanism known as the variable premium which was due to end after the introduction of the European Single Market in 1993.

**Fig. 2:**
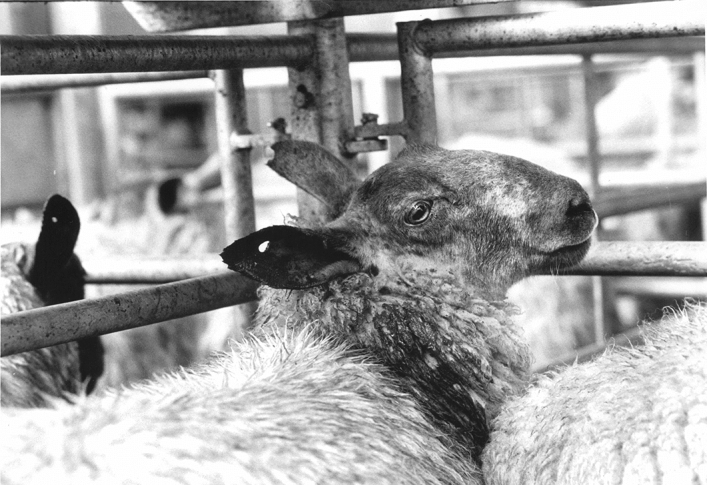
A part of the iconography of live animal export campaigns, photographs like this became a photojournalistic staple in the British press in the 1990s. Photo by Martin Argles, Guardian News Image (1991), reproduced with permission of the Guardian News and Media (GNM 18082020). This image is not covered by the CC BY licence and permission needs to be granted by the Guardian in order to reproduce it

The connections between European agricultural policies—often accused of being overly productionist and leading to wastage of food and animal lives—and these events in France also helped to concentrate public attention in Britain on the conditions in European slaughterhouses and fuelled demands for a ban on live animal exports (Hornsby 1990a). In the face of public pressure, however, the agriculture ministry in Britain was clear in their opinion that banning the trade unilaterally was illegal under EC law. Unsurprisingly then, as Howkins and Merricks (2000, p. 89) comment, the ‘lamb wars’ contributed to “introducing an element of anti-Europeanism” into existing (and ongoing) campaigns protesting the trade. At the same time, readers of the British press became increasingly familiar with what is now a stock trope of both photojournalism and animal activism: caged stock animals on their way to dismal ends, often in Europe (Fig. 2).

Earlier events still helped establish these links. In 1972 the RSPCA and Compassion in World Farming (CIWF) had both launched concerted campaigns against the trade to the Continent. RSPCA inspectors, for instance, traced consignments of sheep from Banbury, Oxfordshire, to slaughterhouses in Europe, documenting welfare abuses along the way. In December that year, CIWF also presented a petition to parliament calling attention to the conditions in continental slaughterhouses, especially in Belgium and France where, CIWF claimed, 85% of sheep were killed without prior stunning (Howkins and Merricks 2000, p. 88; *The *Times 1972; Windsor 1972). While the system of voluntary bilateral welfare ‘assurances’, which was supposed to control how foreign states treated animals of British origin, had been criticised as weak since its inception in 1957, what was putting heat into the issue in 1972 was the UK's  impending accession to the EC. With accession, Treaty of Rome rules on free trade would take effect, making it impossible—or so officials claimed—for individual member states to ban the trade.^7^ These events served to foster cognitive and linguistic links between established ideas about animal cruelty in Europe, and the centralised, constraining nature of European law generally. Officials from successive British governments, despite their support for EU trade policies that benefitted Britain, were also happy to transfer blame for inaction to Brussels, thus avoiding confronting the economic downsides and ideological contradictions that legislative action would necessitate. For example, a government report in 1978 conceded that welfare abuses occurred in European countries, but found that on economic and legal grounds the trade must continue (Norton-Taylor 1978; Phillips 1978). Whether stated or implied, these debates were structured around the assumption, shared by animal advocates, those engaged in the trade and government officials, that the British not only had higher standards but that it was a part of their role to educate Europeans in these matters. *The Times,* for example, noted how the 1978 report accepted as true the “claim made by exporters that the trade enables high British standards to percolate to the less welfare-conscious livestock trades in the other EEC countries" (Clayton 1978).

These patterns continued in the run-in to the creation of the European Single Market in the early 1990s. Existing regulations for animal transportation were due to be eased under the new rules. As the *Guardian* noted, “exports as such” would no longer exist in the Single Market, and “EC rules put live animals in the same class—agriculture produce—as cauliflowers” (Erlichman 1991c). In June 1991, a committee of MPs accused the Agriculture Minister John Gummer of failing to resist EC moves to water down existing domestic rules on the treatment of animals undergoing transportation, claiming in the process that it was an “historical fact that the welfare of animals has been a matter of far greater concern to the British than most continental countries”, and—foreshadowing more recent events—that “the people’s fear of surrendering national sovereignty to an unsympathetic Brussels bureaucracy have been quickened by this issue more than any other” (quoted in Erlichman 1991a). In October, Gummer responded to the pressure by accusing the EC of using lax welfare standards to undermine the competitiveness of British farmers (Erlichman 1991b).

During this period, moreover, the UK was grappling with EC standards on a number of fronts. In particular, it was desperately trying to keep the value of the pound within limits prescribed by the European Exchange Rate Mechanism (ERM), a pre-requisite to joining the Euro. Despite massive expenditure it failed in this endeavour and crashed out of the mechanism. Humiliated, the country was forced to ask for an opt-out at the Treaty of Maastricht in 1992. There was a lesson here: conforming to European standards was a potentially ruinous pastime. While the currency wars predicted economic ruin, in the case of European standards for animal welfare, the matter is more likely moral deterioration and a loss of identity. Concerned about the consequences of further integration, the British novelist and horse welfare campaigner Jilly Cooper told *The Times* that “[w]e love our animals here, and as everyone knows they have a different view on the continent. We should be educating them to be like us, not being forced to fit in with, and assist, their customs” (quoted in Jenkins 1990). While Europe was the immediate concern here, one also senses unease with British multiculturalism more widely. Indeed, while Catholics have long been integrated into national public life, the historical tendency to associate them with animal cruelty is echoed in recent objections to the ‘religious slaughter’ practiced by minorities whose place in the national picture was (and is) less secure, notably Jews and Muslims (see e.g. Klug 1989).

The RSPCA, CIWF and other groups also began campaigning in earnest again at this time. The tactics of these campaigns, which waxed to include mass public demonstrations that in places turned violent (giving rise to media monikers such as the ‘Battle of Brightlingsea’ and the ‘Siege of Shoreham’), need not concern us. Suffice to say that by 1994 pressure was ratcheted up to such an extent that companies engaged in the transportation of animals, including British Airways and two trans-channel ferry companies, felt compelled to end their participation in the trade. Concerned with public order and the cost of policing the protests, various local authorities including the Dover Harbour Board followed suit with bans of their own. In April 1995, the High Court ruled that these bans were illegal under EU law, lending credence to the government claim that it was not sovereign in this matter and could not itself impose a country-wide ban.^8^ In July, the same court confirmed that various practices adopted by the police to control the behaviour of animal hauliers were also illegal under EU law (Hornsby 1995; Joyce and Wain 2014, p. 162). The problem of combining British moral superiority and EU authority were obvious enough. Letters to the Editor in *The Times* focused on correcting the obvious “defect in our constitution”, or declared that “[w]e should be proud that we have an ethical movement in which the people of Britain are leading the world” (Caswell 1995; Gibbon 1995).

In this context, Howkins and Merricks (2000, p. 93) write, there was a noticeable “coming together” of feeling between animal activists and the National Farmers Union (NFU), and even the Ministry of Agriculture, Fisheries and Food (MAFF): “Their agreed point, although never publically made, was growing anti-Europeanism”. While tabloids were (as ever) stocked with slurs concerning the cruelty of foreigners (c.f. O’Neill 2006), the President of the NFU wrote a letter to *The Times* claiming “[i]t is time for decisive action to be taken, both at home and in Europe, to protect animal welfare, and to ensure that our high standards become Europe's high standards” (Naish 1994). Howkins and Merricks comment that it is surprising how few respondents to the Mass-Observation directive they sent out in 1995 on this topic “blamed” the EU or even “foreigners”, given that this was virtually the official position of the MAFF and NFU (Howkins and Merricks 2000, p. 100).^9^ Being unrepresentative however, what is most interesting about the responses reported by Howkins and Merricks is actually how clearly they do in fact articulate animal welfare chauvinism, assumptions of continental cruelty and link these to “anti-Europeanism”. For example, Howkins and Merricks quote one respondent (a 61-year women classroom assistant from London) who wrote: “no live animals should be exported, because as soon as they are, they are out of British control and the French ratbags can set fire to them or do whatever they like with impunity” (Howkins and Merricks 2000, p. 100). Demonstrating concisely how language and imagery from earlier episodes frames that available to describe the present, with its mention of ‘British control’ the example also clearly foreshadows the language of Vote Leave.

This political history and its associated rhetorical reservoir is essential to understanding the power of the issue of live animal export in the context of Brexit. Indeed, fast forward to 2016 and the prospect of disentanglement from Europe, the bosses of Kent Action Against Live Exports (KAALE), a group that organises monthly protests against the trade, explicitly backed leaving the EU in allied terms. Echoing the NFU, the MAFF and others, they stated that membership of the EU has meant that “[i]nstead of their standards coming up to ours, we have to lower ours to accommodate them”, and that many EU countries “have no regard for animal suffering at all” (see statement at Animal Interfaith Alliance 2016). Even critics of the ambition of leaving Europe admit they had a case. In May 2016, for example, Boris Johnson’s father, a pro-EU campaigner and animal welfare advocate, acknowledged that in this area specifically his son’s criticism of the status quo was justified (Webster 2016). To emphasise the point, in August 2016, while the nation was still in the throes of processing the meaning of the referendum result, a day of global action saw protestors (some dressed up as sheep) marching to Westminster to “tell MPs that a vote for Brexit means the UK can and must introduce a ban [on live exports]” (Dalton 2016).

Connected to wider claims concerning how being free of Common Agricultural Policy fetters would allow Britain to raise animal welfare standards, the ease with which live animal export channelled intense emotions towards an identifiable ‘other’ certainly made it attractive to politicians as a symbolic issue. After the referendum, under constant pressure to reach a deal with the EU that could satisfy the UK parliament and thus resolve the clash of constitutional mandates that characterised the crisis, government ministers returned time and again to drink from its well (see e.g. Dathan 2018; Malnick 2017; Villiers 2018; Watts 2018; Webster 2017). Most prominently, Boris Johnson himself weighed in a column in the *Sun.* Citing CIWF sources, Johnson claimed that the UK’s continued participation in EU institutions—notably the customs union, as was then being proposed—would make banning the trade impossible. He reminded his readers that 25 years previously another “Tory [Conservative] government tried to end the trade”, but “Brussels said no—and the government looked foolish”. If Britain compromised on this, he continued, people in this country would continue to be “indignant about the powerlessness of their government”, and animals would continue their “nightmare journeys” to and through Europe. Thus, forcing the languages of national sovereignty and humanity together once again, he concluded that “[i]t is time to take back control. It is time to ban the export of live animals” (Johnson 2018). (Understandably, perhaps, he drew correspondingly little attention to the inevitability that ‘taking back control’ in this case implies constraining the free enterprise of British hauliers and farmers.) After a pledge in the Conservative Party’s election manifesto in 2019, the issue has remained one of the most appealing examples of the benefits of leaving the EU (*BBC * News 2020).

## Power, shame and ‘Caring Britain’

Live animal export occurs all over the world, but in Britain the practice is profoundly associated with received ideas about Europe and Britain’s relationship to it. Indeed, long before the EC/EU, British men and women campaigned passionately about the fate of British horses bound for the butchers of Antwerp and elsewhere in Europe (see e.g. Cronin 2018, pp. 70–79). It was in this context that the phrase ‘a nation of animal lovers’ was (according to Hansard) first used in Parliament. Mentioning the practice of starving horses of water in order to reduce the moisture content of the sausages they would be turned into, Lieut. Commander Fletcher remarked that he felt.

certain that if any member of this House was travelling abroad and he was asked by some foreigner "Are you people in Great ​ Britain humane to animals, are you a nation of animal lovers?", the hon. Member would say at once, that the British are a nation of animal lovers and are peculiarly humane in their treatment of all animals. (HC Deb 5 March 1937)

Complicity with cruelty, such rhetoric suggests, risks forfeiting a place in this construction of the virtuous collective. Certainly, a strategy of shaming delinquent compatriots in nationalist terms is as old as organised animal advocacy in Britain. In 1820, for example, John Lawrence lambasted British hypocrisy in its tolerance of the practice of deliberately laming a horse in one foot so as to even out its stride while simultaneously being in the habit of condemning the cruelty of foreigners: “the disgrace is national and the nation itself responsible”, he argued (cited in Turner 1992, p. 145). In the case of live animal export to the EU, the shame of complicity is likewise palpable. But as much as there is continuity here, there is also a change of tone. With the advent of a supranational political entity and law-making apparatus, a new kind of ignominy gets blended in: the shame of weakness, manifesting as a perceived inability to act freely. The self-perception of weakness in nation states no less than in caste societies has long been noted as a source of dishonour in itself (c.f. Bourdieu 1966; Oprisko 2012; Pitt-Rivers 1966). Thus, according to Boris Johnson, it was the *powerlessness* of their legitimate representatives that made the people of Britain feel indignant, and the *impunity* with which the French ratbags acted that rankled the classroom assistant from London in 1995. This speaks clearly to the idea that powerlessness—the absence of freedom—is a condition of inhumanity.

Of course, the discourse of Brexit has often been said to be infused with postcolonial angst or “nostalgic nationalism” (Franklin 2019; c.f. Koegler et al. 2020). In this case (legal niceties notwithstanding) self-ascription of powerlessness by British elites also serves to divert attention away from their reluctance to regulate and, in this way avoid the contradiction between humanitarian action and professed economic liberalism. However, assuming the position of the colonised is also simply a necessary aspect, rhetorically speaking, of any strategy based on “taking back” power. Indeed, while critics have frequently alleged that Brexit manifested an undercurrent of desire to return to a past in which sovereignty was guaranteed by the authority of the British empire and its navy (see e.g. Barber 2018; Dorling and Tomlinson 2019), perhaps a more prominent construction in the discourse of ‘Brexiteers’ was that of the plucky island nation fighting to regain independence from the empire of Europe (Lane 2019; Saunders 2019). In this light, we can read the captive sheep in Vote Leave’s ad as representing a virtuous but subjugated Britain, forced in its vassalage into sending a horrific tribute to Brussels, and thus bearing both the shame of complicity with cruelty and of subordination. From this perspective, the implied addressee of Vote Leave’s advertisement, summoned by its prominent use of the first-person plural and the direct gaze of the sheep, is simultaneously identified with a struggle for national self-determination and against animal cruelty.

As I have been arguing, then, animal advocates, like government officials, played a role in making such a message intelligible through the strategy of shaming, but also by attacking EU policy where convenient and pointing out where the UK is legally disadvantaged. Indeed, animal advocates unwittingly highlighted the idea that it is Britons who are being symbolically sent to slaughter as much as sheep in a memorable action in 2002 (*BBC *News 2002). Protesting the resumption of the trade to Europe following the end of the foot-and-mouth disease outbreak, activists transformed themselves into sheep and packed tight into a London tube. Using the ‘nightmare journeys’ of human commuters to highlight those nonhuman animals are forced to take, caring British people turned themselves into helpless sheep. But if Britain is to Europe as the sheep is to cruel hauliers licensed by Brussels, then, Vote Leave’s ad suggested, so also does the country, like its indignant citizens, yet retain a hidden integrity. As the Lamb of God’s meekness in death enhanced His virtue, so Britain, despite its present debased condition, can free itself from shame because the true hearts of its people—the nation of animal lovers—actually remain untouched by it. Thus, by conjuring the idea of a ‘Caring Britain’, Vote Leave’s imagery seems simultaneously to hold out a promise of redemption and a return to a prior state of moral and political vigour, or freedom.

## The ‘free movement’ of people and animals

While my term ‘animal welfare chauvinism’ differs from Andersen and Bjørklund’s (1990) concept of “welfare chauvinism” (see above), at times, during the Brexit campaigns at least, it felt at times as though these discursive regimes did interact. Despite Borwick’s claims about their efficacy in online activism, the centrality of the NHS, the benefits system, and immigration were clearly interlinked and powerful issues on the campaign trail (see also Fitzgerald et al. 2020). The systems intended to care for the people in their distress were presented as being in need of care: protecting the NHS, not for the first or last time, became a national political obsession. Putative threats to this symbol of ‘Caring Britain’ included privatisation (or ‘Americanization’), government under funding, lack of nurses following loss of free movement, and ‘health tourism’. Vote Leave’s most infamous pledge was that funds sent to Brussels as a price of EU membership could be redeployed to support the NHS, to a tune of £350 million per week. ‘Leave’ campaigners also regularly emphasised anxiety about benefits fraud and the NHS both being swamped by immigrants, many coming from EU countries, whose enjoyment of these systems allegedly pushed British people to the back of the queue.

The ‘problem’ of freedom movement—whether of ‘goods’ (including animals), workers and residents in the EU, or more specifically Britain’s lack of autonomous control over its borders—thus animated arguments about prioritising care for British people and care for animals sent to Europe for slaughter. In 2014, while then Prime Minister David Cameron was promoting his promise of an in–out referendum if his party won the next General Election, the United Kingdom Independence Party (UKIP) published a campaign poster aimed at highlighting Britain’s defencelessness in the face of immigration from the EU. It featured the ‘iconic’ signifiers of British self-reliance, the White Cliffs of Dover, upon which was superimposed an image of a giant escalator flanked by the slogan: “No border. No control” (see Chapman 2014). Dover, of course, is the closest mainland Britain gets to continental Europe and, consequently, it has always been a site of vulnerability. This vulnerability is strategic, but also moral. The death of migrants attempting the crossing to Britain is one marker of this today. Another is that Dover remains the symbolic centre of the live export trade: countless ferries have departed from what CIWF labelled the “UK capital of the cruel live export trade” in the context of an awareness-raising stunt in which giant images featuring the suffering of sheep being transported to Europe where projected, like UKIPs escalator, onto England’s Europe-facing cliffs (see Compassion in World Farming 2013). The lineage connecting UKIP’s slogan to Vote Leave’s in 2016 is unmistakable. Moreover, read through CIWF’s powerful action, anxieties about control over the forms of life that come or go at Dover can be seen as common themes of very different political campaigns. Both form a part of the genealogy of Vote Leave’s ‘sheep-to-the-slaughter’ advertisement, and together they made it possible and persuasive.

## Conclusion: ‘National culture’ and the re-valuing of values?

This paper suggests that the theme of animal welfare in the context of Brexit is more significant than squabbling over an electoral niche—the “animal lover vote”, as the BBC called it (*BBC *News 2018) (although it was certainly that as well). Rather, it opens a window into how care for animals is mobilised to support different political projects, constitutional arrangements, and ideas about national identity. I have tried to show that one symbolic example, Vote Leave’s ‘sheep-to-the-slaughter’ advertisement, is a product of both chauvinistic and caring impulses that are mutually constitutive and crystallised through discourses formed in relation to contingent historical struggles. These struggles include those intended to secure the welfare of animals and the relative positions of animal advocates, but also other ‘national’ struggles in which elites vie to embody the values of the nation, define its relationship to foreign powers, and secure their positions within its structures of power and government. This may prompt us into re-valuing certain common values, perhaps not so much in terms of their intrinsic worth, but at least in terms of understanding how they function, where their authority comes from, and what they are implicated in.

This brings us finally to the question of ‘a national culture of care’ for animals, the theme of this collection. Evidently, this paper has only addressed *claims about* a putative national culture; that is, rhetorical performances with specific though heterogeneous ends. The extent to which these may be performative in a substantial sense remains open (though, I think, likely). Yet I think this work calls attention to another important point. This is that while culture may be what people (and animals) do every day, and social history may document the culture and practices of ordinary people, there remains a vital component of national life that is decidedly top-down and which needs also to be accounted for empirically and conceptually. National elites, be these politicians or animal advocates with access to powerful means of representation, have significant capacities to shape what citizens take for granted. Even if the British state and national identity were concocted centrally and imposed onto its peripheries, as Colley showed, this does not mean that it was not eagerly adopted at times, often benefitting those who took it on. Correspondingly, that people ‘on the ground’ might identify avidly with ‘the nation of animal lovers’ does not mean that it is not at the same time an elite construction. Reflexively, then, this paper suggests an important point. This is that there is *already* a highly-charged conversation about Britain’s national culture of care for animals going on all around us, and it is often dominated by particular voices; we may intervene into this as scholars only latterly. This is not itself a reason to avoid intervening—but it is a reminder to pause, to listen, and consider the implications of participating.

## Data Availability

This manuscript is comprised of original material that is not under review elsewhere, and that the study(ies) on which the research is based has been subject to appropriate ethical review (Central University Research Ethics Committee (CUREC) of the University of Oxford, Reference Number: SOGE 18A-7.). I have no competing interests—intellectual or financial—in the research detailed in the manuscript to declare.

## Footnotes

1 Photographs from this series of images can be found at the We Animals Archive https://www.weanimalsarchive.org/. See Cronin (2013) for the earliest known use of the image by PETA, campaigners for animal rights.

2 Information about AIQ/*Vote Leave’s* Facebook campaigning, including copies of the original ads in their various iterations, was made available by the House of Commons Digital, Culture, Media and Sport Committee’s (DCMS) “Fake News and Disinformation Inquiry”, via the links provided in a document entitled “Ads supplied by Facebook to the DCMS Committee” (see Facebook 2018). The advertisements can be viewed at: https://web.archive.org/web/20201112103011/http://www.aboutbrexit.uk/vote-leave-fake-news-campaign/.

3 Indeed, there has often been an unacknowledged mercantilism associated with animal welfare in the UK that is at odds with the nation’s professed liberalism. It is widely suggested, for instance, that “buying British” is supposed to be a guarantee of buying meat reared to the highest standards. And “Continentals”, as a House of Commons Library research paper put it, have often alleged that Britain feigns concern for the welfare of exported live animals because it really wants to keep the profits of the abattoir industry onshore (Barclay 1995, p. 1).

4 All newspapers investigated published both pro- and anti-Brexit news stories. Nevertheless, research identified a gradient of bias in the number of stories published that were either pro- or anti-Brexit in orientation. At the top of the list of publishers of 'pro-Remain' stories of the newspapers analysed were the *Daily Mirror, The Guardian* and the *Financial Times*; at the bottom, and therefore most 'pro-Leave' were the *Daily Express*, the *Daily Mail* and the *Sun.* Weighted for circulation and volume, 'pro-Leave' stories by far outweighed pro-Remain during the referendum campaign (Levy et al. 2016, pp. 16–17). The balance of reporting subsequent to the referendum is less clear.

5 Neither is it exclusively British: The United States has also often been styled as 'a nation of animal lovers', and animal protectionism mobilised as a part of it's imperial projects (e.g. Davis 2016). The Third Reich too paid special attention to the supposed union of interests between the German nation and the welfare of animals, notably drawing attention to the ‘problem’ of kosher slaughtering that has echoes today (c.f. Sax 2013).

6 The advertisement can be viewed at: https://web.archive.org/web/20200918164900/https://burniesfoundation.org/media/testing-media/.

7 Actually, the UK government did ban the export of sheep on a temporary basis between 1973 and 1975, but this was never challenged in the European Court of Justice so it is unclear whether it was legal or not. The ban was undone in 1975 (see Barclay 1995, pp. 5–6; Howkins and Merricks 2000, pp. 88–89).

8 This pattern was repeated in 2012 when Thanet District Council attempted to ban live exports from Ramsgate Harbour, after an incident involving a lorry, 43 thirsty sheep, a broken leg, and 41 otherwise lame sheep (*BBC *News 2012).

9 Mass Observation, intended to record impressions of daily life in Britain, produces longitudinal qualitative data in the form of written responses submitted by a non-representative panel of volunteers to regular questionnaires, see http://www.massobs.org.uk/about/about-mo.
